# MOROKOSHI: Transcriptome Database in *Sorghum bicolor*

**DOI:** 10.1093/pcp/pcu187

**Published:** 2014-12-09

**Authors:** Yuko Makita, Setsuko Shimada, Mika Kawashima, Tomoko Kondou-Kuriyama, Tetsuro Toyoda, Minami Matsui

**Affiliations:** ^1^Synthetic Genomics Research Team, Biomass Research Cooperation Division (BMEP), RIKEN Center for Sustainable Resource Science (CSRS), 1-7-22 Suehiro-cho, Tsurumi-ku, Yokohama, Kanagawa, 230-0045 Japan; ^2^RIKEN Advanced Center for Computing and Communication (ACCC), Hirosawa 2-1, Wako, Saitama, 351-0198 Japan

**Keywords:** Database, FL-cDNA, New transcript, NGS, Plant, Sorghum

## Abstract

In transcriptome analysis, accurate annotation of each transcriptional unit and its expression profile is essential. A full-length cDNA (FL-cDNA) collection facilitates the refinement of transcriptional annotation, and accurate transcription start sites help to unravel transcriptional regulation. We constructed a normalized FL-cDNA library from eight growth stages of aerial tissues in *Sorghum bicolor* and isolated 37,607 clones. These clones were Sanger sequenced from the 5′ and/or 3′ ends and in total 38,981 high-quality expressed sequence tags (ESTs) were obtained. About one-third of the transcripts of known genes were captured as FL-cDNA clone resources. In addition to these, we also annotated 272 novel genes, 323 antisense transcripts and 1,672 candidate isoforms. These clones are available from the RIKEN Bioresource Center. After obtaining accurate annotation of transcriptional units, we performed expression profile analysis. We carried out spikelet-, seed- and stem-specific RNA sequencing (RNA-Seq) analysis and confirmed the expression of 70.6% of the newly identified genes. We also downloaded 23 sorghum RNA-Seq samples that are publicly available and these are shown on a genome browser together with our original FL-cDNA and RNA-Seq data. Using our original and publicly available data, we made an expression profile of each gene and identified the top 20 genes with the most similar expression. In addition, we visualized their relationships in gene co-expression networks. Users can access and compare various transcriptome data from *S, bicolor* at http://sorghum.riken.jp.

## Introduction

Sorghum is a highly productive crop, grown for forage, feedstock, fiber and biofuel. It ranks fifth in global cereal production and shows strong environmental stress tolerance against drought, heat, salinity and flooding (Belton et al. 2004). Identifying relevant genes for this stress tolerance and biomass synthesis contributes to improving sorghum traits by genome-guided breeding and facilitates strengthening other crops against various environmental stresses.

In 2009, the *Sorghum bicolor* BTx623 genome was determined as a model species of the Saccharinae and other C_4_ grasses (Paterson et al. 2009). *Zea mays* is the closest relative whose genome sequence has been completely determined (Schnable et al. 2009) and *Oryza sativa* is a closely related and well-studied species in the same grass family (Sakai et al. 2013). Besides genome sequencing, other primary genomic resources are required for further understanding of the stress tolerance mechanism and to enable biomass engineering. We focused on collecting large-scale experimentally validated data sets of transcriptional units, transcription start sites (TSSs) and expression profiles.

A full-length cDNA (FL-cDNA) library and its sequence data provide fundamental information on each transcriptional unit. We can add or fix the annotations that are computationally predicted based on the genome sequence and expressed sequence tags (ESTs). FL-cDNA technology has already been applied to well-studied eukaryotic model organisms (Kawai et al. 2001, Ota et al*.* 2004). In plants, the pioneering work was carried out in *Arabidopsis thaliana* (Seki et al. 2002), and these data are accessible from RARGE (Akiyama et al. 2014) and SABRE2 (Fukami-Kobayashi et al. 2014). Subsequently, the technology has been used in grass species, including *O. sativa* (Kikuchi et al. 2003), *Triticum aestivum* (Ogihara et al. 2004, Kawaura et al. 2009), *Hordeum vulgare* (Sato et al. 2009, Matsumoto et al. 2011), *Z. mays* (Soderlund et al*.* 2009) and *Brachypodium distachyon* (Mochida et al. 2013).

In Arabidopsis, several new useful resources have been constructed based on FL-cDNA information. An example is the FL-cDNA Over-eXpressor gene (FOX) hunting system that expresses functional FL-cDNAs individually in plants (Ichikawa et al. 2006, Kondou et al. 2009). Around 10,000 normalized FL-cDNAs were transformed into Arabidopsis that resulted in various phenotypes and opened up new avenues of research (Fujita et al. 2007). To develop sorghum research further, we constructed a normalized FL-cDNA library (manuscript in preparation) and created a transcriptome database.

FL-cDNAs also provide accurate TSSs. Since transcription factor-binding sites are located around TSSs, accurate information on TSSs increases understanding of transcriptional regulation and allows analysis of the associated network. This database includes around 35,366 FL-cDNA 5′ reads mapped by Sanger sequencing and 20,626 newly annotated TSSs.

In addition to the correct annotations of the transcriptional units from the FL-cDNAs, the expression profiles from RNA sequencing (RNA-Seq) analysis provide us with further transcriptome information, such as tissue and developmental specificity, and co-transcription. We first focused on sugar to starch metabolism and applied RNA-Seq analysis to spikelets at the anthesis stage, and to seeds that accumulated starch, using the stem as a control (manuscript in preparation).

Genes that are co-transcribed by the same transcription factors or that are involved in functionally related biological pathways show similar expression patterns. They are often classified into functionally related groups, and co-expression networks can be established. Previously, microarrays took the central role in co-expression analysis (Shakoor et al. 2014). However, the development of next-generation sequencing (NGS) and RNA-Seq analysis has seen these technologies take the lead, as they allow higher gene coverage than microarrays in Arabidopsis (Obayashi et al. 2014). In addition to our original data, we used 23 samples that were published in four studies (Dugas et al. 2011, Davidson et al*.* 2012, Yazawa et al*.* 2013, Gelli et al*.* 2014). Including our data, a total of 52 replicates from 26 samples were used to plot expression profiles for each gene. We also display the top 20 genes that are most closely related, which are predicted to be co-regulated and to share function, and show co-expression networks.

## Results

### FL-cDNA clones and their Sanger sequence annotation

We constructed a normalized FL-cDNA library of *S. bicolor* (L.) Moench from eight growth stages including anthesis and seed set (Table 1), and obtained 38,981 high-quality Sanger sequence reads after quality control (manuscript in preparation). For the 5′ end sequences, we obtained 37,607 sequences with a mean length of 714.9 bases (the maximum was 900 bases and the minimum was 100 bases) and we mapped them against Sbicolor_255 (Goodstein et al. 2012) using GMAP mapping tools (Wu et al. 2005). Newly identified clones that had no gene annotation in Sbicolor_79 were Sanger-sequenced from the 3′ end to determine full-length transcripts. We obtained 1,374 sequences with a mean length of 565.2 bases (the maximum was 823 bases and the minimum was 105 bases). In total, 814 contigs were connected from both ends and they mapped to 255 genes (Table 2).

**Table 1:** Sampling tissue and stage details for FL-cDNA and RNA-Seq data

Category	Sample name*^a^*	Stage
FL-cDNA	Aerial tissues 1	7 d after sowing
	Aerial tissues 2	14 d after sowing
	Aerial tissues 3	30 d after sowing
	Aerial tissues 4	60 d after sowing
	Aerial tissues 5	90 d after sowing
	Aerial tissues 6	150 d after sowing (at the time of anthesis)
	Aerial tissues 7	165 d after sowing
	Aerial tissues 8	180 d after sowing

RNA-Seq	Spikelet	150 d after sowing (at the time of anthesis)
	Seed	165 d after sowing
	Stem	150 d after sowing

*^a^* Aerial tissues contain leaves, stems and panicles.

**Table 2:** FL-cDNA sequence resources in *S. bicolor*

Category	No.
Partial full-length cDNA sequences	38,981
Sanger 5′ ESTs	37,607
Sanger 3′ ESTs	1,374
Total sequences mapped onto the genome	36,700
No. of genes (loci) annotated by our data	10,811
Overlapped known	9,566
Partially overlapped known genes	650
Unknown (newly identified)	272
Antisense transcripts	323
Full-length cDNA reached from both ends (contigs)	814
Full-length cDNA reached from both ends (genes)	255

A total of 38,981 FL-cDNA ESTs were mapped to 9,566 genes in Sbicolor_255. Around one-third (29.0%) of the known genes generated FL-cDNA clones and the transcription start sites (TSSs) and/or transcription termination sites were confirmed. With these sequences, we successfully improved the structural gene annotations. We updated the untranslated regions (UTRs) of 8,873 genes, re-annotated 80 genes into 40 fused genes, and identified 272 putative novel genes, 323 antisense transcripts and 1,672 candidate isoforms (manuscript in preparation).

### Annotation of transcription start sites based on FL-cDNAs

**Fig. 1:**
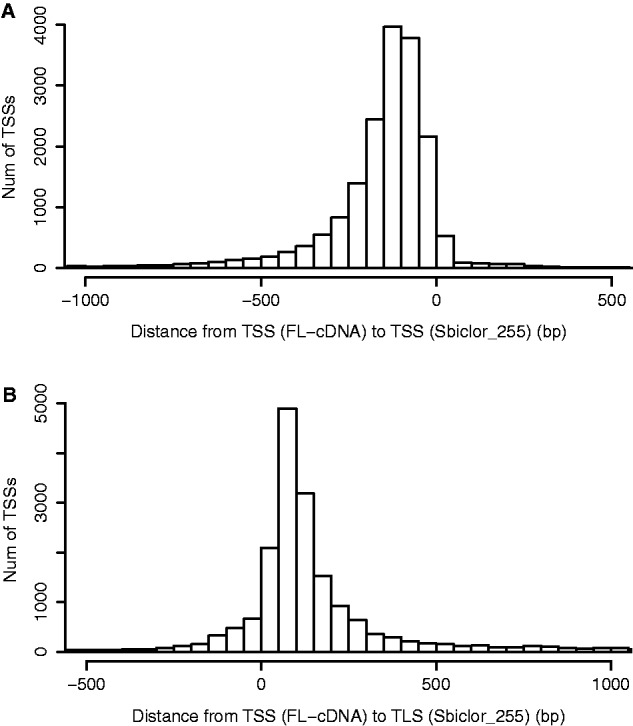
(A) Distance from our identified transcription start site (TSS) to the nearest transcription start sites in the Sbiclor_255 annotation. (B) Distance from our identified transcription start site to the translation start site (ATG).

Since transcription factor-binding sites are located around TSSs, it is very important to define precise TSSs. Currently 35,910 unique positions of TSSs are annotated in the Sbicolor_255 data set. These are mainly estimated using ESTs. However, our FL-cDNA data located 20,680 unique positions of TSSs, and only 54 of them were the same as with Sbicolor_255. We checked the distance from our annotated TSSs to the Sbicolor_255 TSS data (Fig. 1A). From our TSS data, the UTR length of most (94.2%) turned out to be shorter than the Sbicolor_255 annotations. Fig. 1A suggests that Sbicolor_255 predicts longer transcripts than the observed transcripts.

We analyzed the –50 bp to +10 bp sequence motif around the TSSs that annotated at the same position in both Sbicolor_255 and our FL-cDNA data. The ‘[C/T][C/T][G/C]’ motif can be detected at –2 to +1 bp with low frequency.

Fig. 1B shows the distribution of the distances from the TSS to the nearest translation start site (TLS). In approximately 70.4% of the genes this was found to be within 200 bp and suggests that sorghum has relatively compact 5′ UTRs like Arabidopsis (75.5% of the TSSs located within 200 bp of the TLSs).

### *Sorghum bicolor* genome re-annotations with FL-cDNA ESTs and publicly available ESTs

We downloaded 203,816 publicly available ESTs and combined them with our 38,981 FL-cDNA ESTs and the re-annotated sorghum genes using the PASA (Program to Assemble Spliced Alignments) pipeline. They were assembled into 36,776 sequences. These were mapped to 18,374 genes, and 610 protein sequences were modified. The numbers of gene model updates are shown in Table 3. In the PASA pipeline, our 5′ ESTs are not distinguished from other ESTs, and the TSS information was not fully utilized. For this reason, the number of reduced 5′ UTRs is fewer than expected and no novel genes are added. Additionally, 958 alternative splicing isoforms are annotated.

**Table 3:** Number of gene model updates by the PASA pipeline using 242,797 ESTs

Category	No.
UTR extension	18,137
Altered protein sequences	309
Stitched into gene structure	274
Merging multiple genes	29
Total*^a^*	18,601

*^a^* Some models are in multiple classes.

### Tissue-specific RNA-Seq sequence analysis

**Fig. 2:**
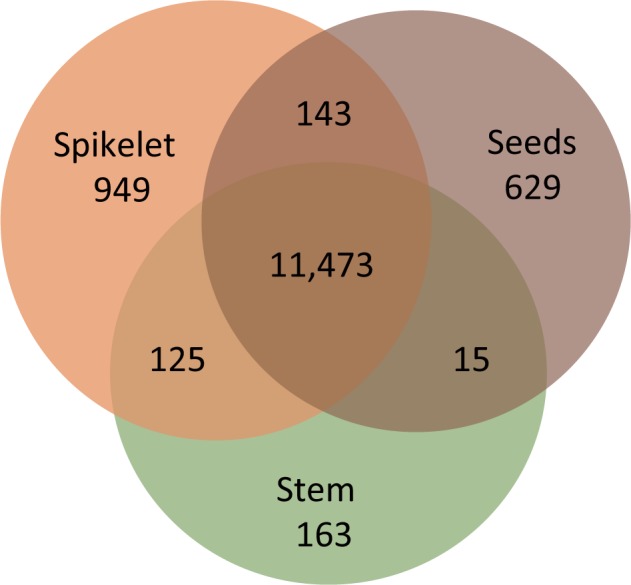
Venn diagram showing the tissue-specific gene expression profiling in spikelets, seeds and stems. In this figure, over five FPKM values are regarded as expressed, and less than one FPKM value is considered as non-expressed.

The *S. bicolor* BTx623 strain whose genome has been determined is called grain sorghum and it accumulates starch in the grain. To compare the genes expressed during starch accumulation, we prepared RNA from spikelets at the anthesis stage and from seeds. For control samples, we took RNA from the stem at the same time as the spikelets were harvested (Table 1). We applied a next-generation RNA sequencing approach to these three samples in triplicate. In total, 94.3% (31,147/33,032) of sorghum genes were expressed [FPKM (fragments per kilobase of transcript per million mapped reads) >0] in at least one of our RNA-Seq data. We also confirmed the expression of 272 of the genes newly identified from the FL-cDNAs. As a result, the expression of 192 genes (70.6%) was also confirmed with our RNA-Seq data (Table 4). In addition, we checked the tissue specificity of the genes in our samples. In order to extract only the genes that are clearly expressed, we defined the expressed FPKM value as ≥5. Similarly, in regard to defining the slightly and partially expressed genes as non-expressed, we set the non-expressed FPKM value < 1. Using these criteria, we identified 949 genes as spikelet specific, 629 as seed specific and 163 as stem specific. A total of 11,473 genes were expressed in all three (Fig. 2).

**Table 4:** Summary of overlapped genes between FL-cDNA and RNA-Seq data

	No. of detected genes with FL-cDNA	No. of expressed genes in RNA-Seq	No. of shared genes with RNA-Seq and FL-cDNA*^a^*
Known genes*^b^*	9,837	22,824	9,326 (94.8%)
Newly detected genes	272	2,592	192 (70.6%)
Antisense	323	223	53 (16.4%)

*^a^* The values in parentheses are the percentage of overlapped expressed genes in both FL-cDNA and RNA-Seq.

*^b^* Known genes include partially overlapped transcripts.

Users can access both the expression results and the link to GBrowse from the gene page.

### MOROKOSHI database function and its web interface

**Fig. 3:**
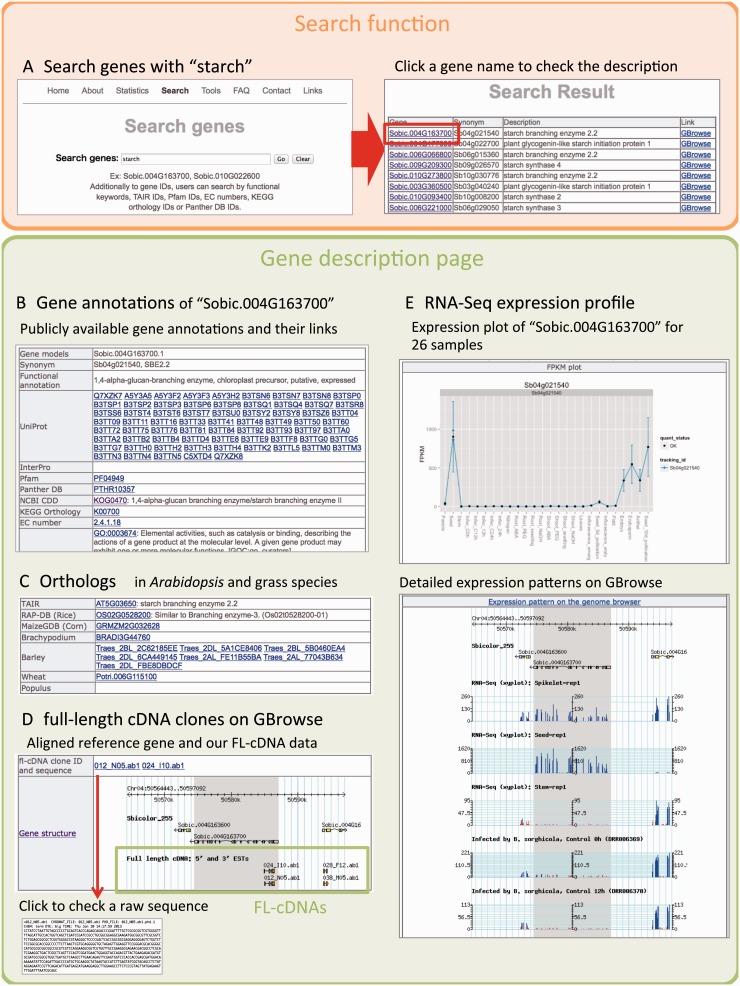

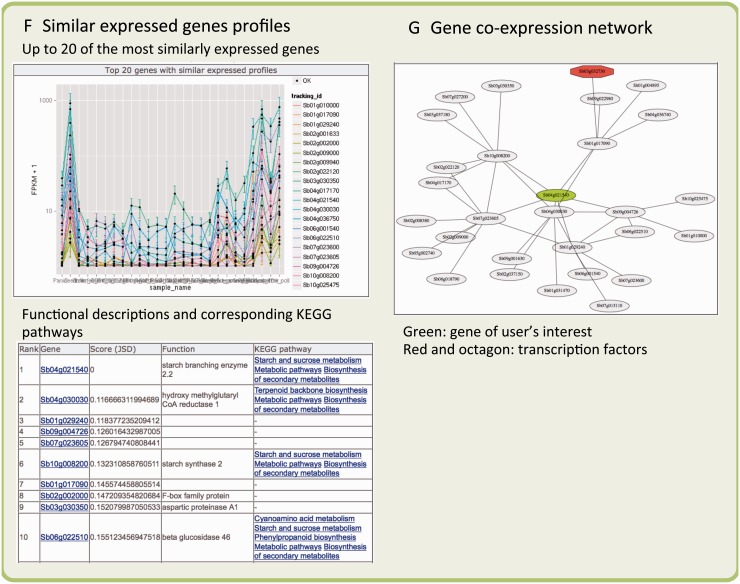
The web interface for the MOROKOSHI database. (A) Search function, retrieve with a keyword of ‘starch’ and its result page. (B) Gene annotation for the Sobic.004G163700 gene from a variety of public databases. (C) Orthologous genes in Arabidopsis, rice, corn, *Brachypodium*, barley, wheat and *Populus*. (D) Mapping result of FL-cDNA clones and their raw sequence data. (E) Expression profile of Sobic.004G163700 using 26 samples of RNA-Seq data and their mapping results on GBrowse. (F) Up to 20 genes with expression most similar to Sobic.004G163700. (G) Gene co-expression network of Sobic.004G163700 and similarly expressed genes.

We provide experimentally validated TSSs that have been derived from FL-cDNAs and from the results of gene co-expression analysis based on our original and publicly available RNA-Seq data. The information is organized for each gene, and users can retrieve their gene of interest by its gene ID or functional keyword(s) (Fig. 3A). At the top of the gene description page, annotation information from a variety of public databases, such as UniProt (UniProt Consortium. 2014), InterPro (Hunter et al*.* 2012), Pfam (Finn et al*.* 2014), PantherDB (Mi et al. 2013), NCBI CDD (conserved domains) (Marchler-Bauer et al*.* 2013), KEGG orthology (Kanehisa et al. 2014), EC number, and GO (gene ontology) (Blake et al*.* 2013), is available (Fig. 3B). As shown in Fig. 3C, users can check orthologous genes of Arabidopsis, *O. sativa*, *Z. mays*, *Brachypodium* and *Populus* that are extracted from the GRAMENE database (Monaco et al. 2014). In the next section, mapping results of FL-cDNA clones are available on GBrowse and it contains a link to raw sequences (Fig. 3D). This FL-cDNA information helps to identify experimentally validated TSSs (not computational predictions). In Fig. 3E, the expression profile of each gene is visualized with a FPKM plot, and mapping results of all 26 RNA-Seq data are available on GBrowse. Below the expression pattern of the gene there is a list of the top 20 genes that are most similarly expressed. These are candidates as functionally related genes. We also describe the corresponding KEGG pathway for each gene (Fig. 3F). At the end of the gene description page (Fig. 3G), we visualize the co-expression network as an overview of expression similarity of the gene of interest (see the Materials and Methods).

By clicking a different tab (Tools), users can also perform a BLAST search against our original Sanger-sequenced FL-cDNA 5′/3′ sequences, nucleotide coding sequences (CDS) and peptide sequences.

### Implementation of the website

The MOROKOSHI website is currently running on Linux (Ubunts 14.04) with the following environments: Apache HTTP server (ver. 2.4.7), PHP (ver. 5.5.9), Perl (ver. 5.18.2) and Python2.7. As a relational database management system, we set up the MySQL (ver. 5.5.40) for faster data access from the genome browser of GBrowe (ver. 2). Genes description pages are generated as static web pages.

## Conclusion

In this database, users can access predominantly two types of transcriptome data; correct TSSs and structural gene annotation based on approximately 40,000 FL-cDNAs, and expression profiles from RNA-Seq analysis.

We first sequenced approximately 40,000 sorghum FL-cDNA reads. These covered around one-third of the known genes and suggested 272 new genes and 1,672 new isoforms. Based on these data, we constructed a sequence database of sorghum FL-cDNAs that is useful for the correct annotation of predicted transcriptional units and gene products. In this database, we also have >20,000 newly annotated TSSs, and these precise TSSs will help with promoter analysis. The motif findings in particular will be useful for transcription factor-binding sites. A total of 97.8% of the 272 genes newly annotated by FL-cDNA assembly have also had their expression confirmed with our original RNA-Seq and/or the publicly available RNA-Seq data.

The second part of this database is aimed at understanding the functional relationships between genes, their transcripts and regulatory proteins using expression profiles. We have combined three tissue-specific RNA-Seq data sets and other publicly available data to produce expression profiles. Based on the maximum available RNA-Seq data, we aim to facilitate users’ research by showing genes that are co-expressed especially for functionally unknown and/or sorghum-specific genes.

## Materials and Methods

### Sequencing and mapping of FL-cDNA clones

We constructed a normalized FL-cDNA library from the aerial tissues of panicles, leaves and top internode stems at eight time points (Table 1). The plants used for RNA extraction were grown in soil in a greenhouse. Tissues were collected from each developmental stage and ground in liquid nitrogen. Total RNAs from each tissue were extracted using the SDS/phenol method followed by LiCl purification (Shirzadegan et al*.* 1991), mixed and used for making a cDNA library. The cDNA library was Sanger sequenced from the 5′ end (manuscript in preparation). A total of 37,619 sequences were generated that had a Phred quality of ≥20, and 12 sequences were discarded after using SeqClean (http://sourceforge.net/projects/seqclean/), which validates and trims DNA sequences. We also applied 3′ end Sanger sequencing to the cDNA clones that contained newly identified genes and antisense transcripts. Sequence quality controls were carried out in the same way as for the 5′ end sequences. After the sequencing and quality controls, we mapped 38,981 FL-cDNA ESTs to the Sbicolor_255 genome using the GMAP mapping software (Wu et al. 2005) in the PASA (Program to Assemble Spliced Alignments) pipeline (Haas et al*.* 2003) with default parameters. Our FL-cDNA sequence data were submitted to the DNA Data Bank of Japan (DDBJ; PRJDB3280).

### FL-cDNA annotation with publicly available ESTs by PASA

In addition to our 38,981 FL-cDNA 5′ and 3′ end sequences, we downloaded 203,816 ESTs from the PlantGDB (Duvick et al. 2008) and re-annotated transcripts of *S. bicolor*. Following previous FL-cDNA research (Campbell et al*.* 2006, Mochida et al*.* 2013), we applied a total of 242,797 ESTs to an annotation pipeline of PASA with default parameters. In this pipeline, we used two mapping software programs; gmap and blat. PASA automatically combined these results and re-annotated the current structural gene annotations.

### Analysis of RNA-Seq sequences

We prepared RNA samples from spikelets and stems from plants aged 5 months at the anthesis stage and from seeds from plants 2 weeks older (Table 1). All samples were grown in the soil, and detailed RNA samplings will be described in Shimada et al*.* (manuscript in preparation). We performed directional RNA-Seq with the HiSeq2000 Illumina. The read length was 50 bp of single reads and sequence read data were submitted to the DNA DDBJ (PRJDB3281). For sequence quality control, we used the FASTX-Toolkit (http://hannonlab.cshl.edu/fastx_toolkit/). First, we trimmed base pairs with a Phred quality of ≤20 from the 3′ end of each sequence and discarded the sequence when it was shorter than 30 bp in length. Next, if 20% of a sequence had a Phred quality of ≤20 then that sequence was discarded. Sequences that passed these two filters were mapped with TopHat v2.0.11, assembled and compared using Cufflinks v2.2.0.

### Expression profile analysis

In addition to our original RNA-Seq samples, we downloaded 23 samples of 43 publicly available RNA-Seq data from four studies (Dugas et al. 2011, Davidson et al*.* 2012, Yazawa et al*.* 2013, Gelli et al*.* 2014). All data were trimmed, filtered, mapped and assembled in the same way as our data. After the assembly, we used an R package of CummeRbund that is designed to assist and simplify the task of analyzing Cufflinks RNA-Seq output (Trapnell et al. 2012). With CummeRbund, we generated expression profile figures against each gene and calculated the top 20 genes with similar expression profiles.

### Genome Browser and BLAST search

All of the FL-cDNA ESTs, downloaded ESTs, our original RNA-Seq data and publicly available RNA-Seq data are shown on the Generic Genome Browser (GBrowse2) (Stein et al. 2002) with sorghum genome annotations released by Phytozome v10 (Goodstein et al. 2012). The BLAST search function is also available in the database. Our total FL-cDNA EST data are provided as the BLAST database.

### Functional annotation

In our database, users can access functional annotations of Sbicolor_255, UniProt, InterPro, Pfam, Panther DB, NCBI CDD, KEGG Orthology and EC numbers. Cross-link data are derived from Sbicolor_255 and GRAMENE (Monaco et al. 2014). Also, orthologous information of *A. thaliana*, *O. sativa* L. ssp. *japonica*, *Z. mays*, *B. distachyon* and *Populus trichocarpa* is downloaded from GRAMENE BioMart (Spooner et al*.* 2012).

## Funding

This research is conducted under the research program of RIKEN Biomass Engineering.

## Abbreviations

DB: database
EST: expressed sequence tag
FL-cDNA: full-length cDNA
FPKM: fragments per kilobase of transcript per million mapped reads
NGS: next-generation sequencing
PASA: Program to Assemble Spliced Alignments
RNA-Seq: RNA sequencing
TSS: transcription start site
UTR: untranslated region
